# Foxm1 is a critical driver of TGF‐β‐induced EndMT in endothelial cells through Smad2/3 and binds to the Snail promoter

**DOI:** 10.1002/jcp.27583

**Published:** 2018-10-30

**Authors:** Shuai Song, Rui Zhang, Wei Cao, Guojian Fang, Yi Yu, Yi Wan, Chuanhui Wang, Yigang Li, Qunshan Wang

**Affiliations:** ^1^ Department of Cardiology Xinhua Hospital, School of Medicine, Shanghai Jiao Tong University Shanghai China; ^2^ Department of Ultrasound Xinhua Hospital, School of Medicine, Shanghai Jiao Tong University Shanghai China; ^3^ Department of Geriatric Shanghai Ninth People's Hospital, Shanghai Jiaotong University School of Medicine Shanghai China

**Keywords:** cardiac fibrosis, EndMT, Foxm1, snail, TGF‐β1

## Abstract

Endothelial‐to‐mesenchymal transition (EndMT) was first reported in heart development. Recent studies have shown that EndMT also occurs in the progression of cardiac fibrosis. Herein, we demonstrated a critical role of the Forkhead Box M1 (Foxm1) transcription factor in transforming growth factor beta (TGF‐β)‐induced EndMT in endothelial cells (ECs) and a possible underlying molecular mechanism. Foxm1 was induced in ECs following TGF‐β stimulation. Using both pharmacological and molecular approaches to inhibit Foxm1 function can attenuate the TGF‐β‐induced EndMT and cell migration. In contrast, lentivirus‐mediated overexpression of Foxm1 allowed EndMT to proceed despite the absence of TGF‐β in ECs. Moreover, we found that the activation of the Smad2/3 signaling pathway and EndMT‐related transcription factors played important roles in the pathogenesis of Foxm1‐mediated EndMT. Further analysis revealed that Foxm1 bound to and increased the promoter activity of the *Snail* gene encoding a critical transcriptional regulator of EndMT. In conclusion, our results identify FOXM1 as a driver of TGF‐β‐induced EndMT and underscore the therapeutic potential of targeting FOXM1 for cardiac fibrosis.

## 1 INTRODUCTION

Heart failure (HF) is a devastating disease that remains a leading cause of death worldwide as well as a major socioeconomic burden on society. An underlying morphological correlate of HF is cardiac fibrosis (CF; Gonzalez, Schelbert, Diez, & Butler, 2018). However, specific antifibrotic therapies are not currently available in the clinic (Vasan & Benjamin, 2001). The predominant cellular mediators of CF in the heart are thought to be fibroblasts, but until recently, little was known about the source of the newly formed fibroblasts (Zeisberg & Kalluri, 2010). Potential sources included resident fibroblasts, circulating cells originating from the bone marrow, and epithelial cells contributing to fibroblast accumulation through the epithelial–mesenchymal transition (EMT; Iwano et al., 2002).

Endothelial–mesenchymal transition (EndMT) is a form of EMT, in which endothelial cells (ECs) lose their specific markers, such as VE‐cadherin and CD31, acquire a mesenchymal or myofibroblast phenotype and express mesenchymal cell products, such as vimentin, α‐smooth muscle actin (α‐SMA), and fibroblast‐specific protein‐1 (FSP1; Li et al., 2018). EndMT was originally observed during the heart development, and recent studies have suggested its role in pathological settings including CF (Xu et al., 2015a) and pulmonary arterial hypertension (PAH; Hopper et al., 2016). EndMT can be induced by hypoxia, high glucose, transforming growth factor‐β (TGF‐β), inflammation, and radiation (Xu et al., 2015b; Zeisberg et al., 2007). Studies have shown that TGF‐β stimulates EndMT through the Smad2/3 signaling pathway, and this stimulation is essential for increasing the expression of cell‐adhesion‐suppressing transcription factors (TF), such as Snail, Twist, and Slug, which may be potential targets in CF (Medici, Potenta, & Kalluri, 2011).

Forkhead box M1 (Foxm1) is a transcription factor best‐recognized as a master regulator of physiological and pathological processes, including cancer (Aytes et al., 2014), diabetes mellitus (Shirakawa et al., 2017), and fibrosis‐related disease (Penke et al., 2018). Among its isoforms, Foxm1b (hereafter designated simply as FOXM1) has been studied most extensively and is considered to be able to activate the expression of multiple target genes critical for normal cell proliferation, survival, and self‐renewal as well as cancer initiation, progression, and drug resistance (Koo, Muir, & Lam, 2012; Wang et al., 2018). A growing body of recent evidence has emphasized the potential roles of Foxm1 in organ fibrosis, such as pulmonary fibrosis (Balli et al., 2013) and CF (Sato et al., 2017). However, the underlying molecular mechanisms of Foxm1 in cardiac fibrosis are not clearly elucidated, especially the role of Foxm1 in EndMT, which remains unexplored. Here, we investigated the role of Foxm1 in TGF‐β‐induced EndMT in ECs and clarified a possible molecular mechanism. The data presented in this report provide new insights into the regulation of FOXM1 in EndMT‐induced CF and provides important experimental evidence supporting Foxm1 as a potential therapeutic target for cardiac fibrosis.

## 2 MATERIALS AND METHODS

### 2.1 Cell culture

Primary human umbilical vein endothelial cells (HUVECs) were obtained from human umbilical cord veins and maintained in a humidified atmosphere at 37°C in 5% CO_2_ using the EGM‐2 bullet kit (Lonza, Basel, Switzerland). Cells between passages two and six were plated on vitronectin‐coated dishes, and used in each experiment. Human microvascular endothelial cells (HMECs) were purchased from Lonza and maintained in EGM‐2 media with supplements as previously described (Sabbineni, Verma, & Somanath, 2018). Mouse aortic endothelial cells (MAECs) were separated from abdominal aorta of C57BL/6J mice (8–12 weeks of age) as previously described. (Kobayashi, Inoue, Warabi, Minami, & Kodama, 2005) To induce EndMT, recombinant TGF‐β1 (10 ng/ml, Peprotech, Rocky Hill, New Jersey) and TGF‐β2 (10 ng/ml, Peprotech) in serum‐free medium was used to treat the cells for 48 hr.

### 2.2 Quantitative real‐time PCR

Total RNA was extracted from cultured cells using a TRIzol reagent (Invitrogen). Reverse transcription was performed using a first strand complementary DNA (cDNA) reverse transcription kit, and real‐time polymerase chain reaction (RT‐PCR) was performed using a SYBR Green/ROX qPCR Master Mix kit according to the manufacturer's instructions (Takara, Otsu, Japan). The relative quantification was determined using the ΔΔCt method with *GAPDH* as a reference gene. All primer sequences are shown in Supporting Information Table 1.

### 2.3 Western blot analysis

Proteins from cultured cells were extracted with radioimmunoprecipitation assay (RIPA) lysis buffer (Thermo Scientific, Waltham, MA #89901) and Protease and Phosphatase Inhibitor Cocktail (Thermo Scientific #78447) and quantified by the bicinchoninic acid (BCA) protein assay kit (Thermo Scientific #23225). Then the proteins were separated on sodium dodecyl sulfate‐polyacrylamide gel electrophoresis (SDS‐PAGE) and transferred to a polyvinylidene fluoride (PVDF) transfer membrane. After being blocked, the membranes were incubated with primary antibodies against Foxm1 (CST #20459), VE‐cadherin (Abcam, ab33168), CD31 (Abcam, ab24590), α‐SMA (Abcam, ab21027), vimentin (Abcam, ab92547), FSP1 (Abcam, ab124805), Snail (Abcam, ab216347), and followed by appropriate secondary antibodies. The proteins were visualized using Electrochemiluminescence (ECL) reagents. Blots were quantified by densitometry using ImageJ (NIH Image, Bethesda, Maryland).

### 2.4 Immunofluorescence staining

After different stimulations, primary HUVECs were fixed in 4% paraformaldehyde for 15 min and permeabilized with 0.1% Triton‐X100 in phosphate‐buffered saline (PBS) for 10 min. After blocking with 5% bovine serum albumin (BSA) for 1 hr at room temperature, the cells were incubated with primary antibody Vimentin (Abcam, ab45939) and CD31 (Abcam, ab24590) at 4°C overnight. The cells were then incubated with 4′,6‐diamidino‐2‐phenylindole (DAPI) and Alexa Fluor® 488‐conjugated goat anti‐rabbit IgG H&L for vimentin and Alexa Fluor® 647‐conjugated goat anti‐mouse IgG H&L for CD31 at room temperature for 1 hr. The cells were visualized and photographed under a Zeiss fluorescence microscope.

### 2.5 Knocking down of Foxm1 by lentiviral short hairpin RNA (shRNA)

The following Foxm1‐targeted short hairpin RNAs (shRNAs) were designed and synthesized by Genechem Co. Ltd (Shanghai, China). The recombinant lentivirus of small interfering RNA (siRNA) targeting Foxm1 (sh‐Foxm1) and control lentivirus (sh‐NC) were commercially prepared. Briefly, a lentivirus transfer vector (GV118) was constructed. The vector contained an ampicillin resistant gene and an enhanced green fluorescent protein gene. Expression of shRNA was driven by a U6 promoter. The packaging of viruses was performed by transient transfection of 293T cells with a transfer plasmid and three packaging vectors: pGC‐LV, pHelper 1.0, and pHelper 2.0. Three days after the transfection, lentiviral particles were collected, filtered, and concentrated by ultracentrifugation at 50,000 *g* for 2.5 hr at 4°C. The lentiviral‐shRNA was transfected into primary HUVECs at a final concentration of 50 ×PFU/cell (after cells had grown to 30–50% confluence). After 45 hr, the knockdown efficiency of shRNA was evaluated by qRT‐PCR and western blot analysis. The targeting sequence of the shRNA: (a) CCGGCGCCGGAACATGACCATCAAACTCGAGTTTGATGGTCATGTTCCGGCGTTTTT; (b) CCGGGCCAATCGTTCTCTGACAGAACTCGAGTTCTGTCAGAGAACGATTGGCTTTTT; (c) CCGGGCCCAACAGGAGTCTAATCAACTCGAGTTGATTAGACTCCTGTTGGGCTTTTT.

### 2.6 Inducible FOXM1 expression in HUVECs

The tetracycline‐inducible lentiviral pCW57.1‐HA‐FOXM1b was a gift from Adam Karpf (Addgene plasmid #68811). The plasmid was sequence verified. Replication‐deficient lentivirus expressing Dox‐inducible FOXM1 was produced by transient transfection of 6.0 μg psPAX2 (Addgene: 12260), 2.0 μg pMD2.G (Addgene: 12259), and 8.0 μg transfer plasmid into HEK293T cells in a 10‐cm dish with Lipofectamine 2000 reagent (Life Technologies), according to the manufacturer's instructions. Viral supernatants were collected at 48 hr, passed through a 0.45‐μm filter, and titered by serial dilution with puromycin (Life Technologies) selection and colony formation. The highest dilution producing drug selected colonies were used to transduce primary HUVECs in the presence of polybrene (4 μg/ml, Sigma), and 0.5 μg/ml puromycin was introduced 48 hr postinfection. Cells were seeded in 6‐well plates and the next day media was changed with or without doxycycline (Sigma) to induce transgene expression. Media with or without doxycycline was changed every 24 hr. After 48 hr, cells were prepared for total RNA and protein extractions.

### 2.7 Chromatin immunoprecipitation assays

After HUVECs had been treated with TGF‐β1 (10 ng/ml) or vehicle for 48 hr, protein–DNA complexes were cross‐linked using 1% formaldehyde (Thermo Scientific) for 10 min at room temperature, and the reaction was quenched with 125 mM glycine. Cells were washed with PBS and lysed by incubation in immunoprecipitation (IP)‐buffer. Chromatin was sheared by sonication (Bioruptor, Diagenode) to obtain an average size of 500–1,000 bps. Protein–DNA complexes were immunoprecipitated overnight using antibodies selective for rabbit polyclonal Foxm1 (CST, #20459), rabbit IgG (Santa Cruz, sc‐2072) served as negative controls. The immune complexes were adsorbed with protein A agarose beads (Invitrogen), Immunoprecipitates were washed, eluted, and crosslinks were reversed overnight. The next day, samples were clarified by phenol:chloroform:isoamyl alcohol extraction. IP and non‐IP DNA (input) were analyzed by real‐time PCR. As a positive control for IP‐analysis, primers directed against rat GAPDH promoter were used. PCR are using the following primers: 5′‐TCTTACCCCGGGCCTTTCCCCTCG‐3′ and 5′‐CCGCTCGAGTGGCCAGAGCGACCTAG‐3′. Enrichment of specific promoter regions after IP was calculated as fold induction over IgG.

### 2.8 Migration assay

Cell migration was assessed using transwell chambers (8 μm pore size; Corning, Corning, New York). The samples containing 1 x 10^5^ cells were resuspended in EGM‐2 serum‐free medium and loaded into the upper chamber. The chambers were incubated for 24 hr with complete culture medium added in the lower chamber. Nonmobile cells were removed, and the chambers were stained with crystal violet. Five randomly selected fields were counted under an inverted light microscope.

### 2.9 Statistical analysis

Data were presented as means ± SEM. All experiments were repeated at least three times. Statistical analysis was performed with SPSS software (v19.0, Chicago, Illinois). Comparisons among groups were made using one‐way analysis of variance or the Student's *t* test. The *p* values < 0.05 were considered significant.

## 3 RESULTS

### 3.1 TGF‐β induces EndMT in cultured HUVECs

**Figure 1:**
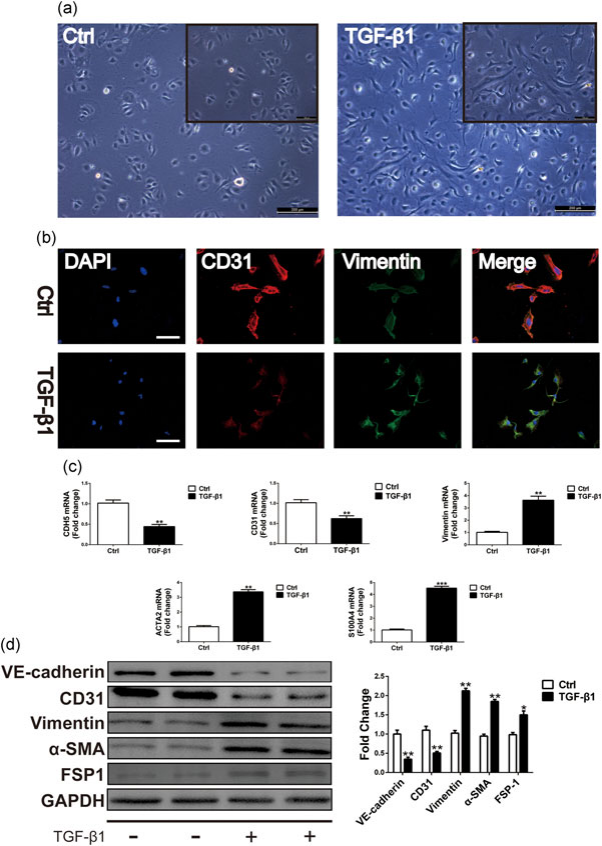
TGF‐β1 induces EndMT in cultured primary HUVECs. (a) Representative phase light microscopy images showing typical cobblestone morphology of control (Ctrl) versus a monolayer including cells with a fibroblast‐like phenotype in primary HUVECs following exposure to TGF‐β1 (10 ng/ml) 48 hr. Scale Bar = 200 μm. (b) Cells were analyzed by immunofluorescence for the expression of endothelial cells (ECs) marker CD31 (red) and fibroblasts marker vimentin (green) and nuclei (DAPI: blue). Scale Bar = 100 μm. (c) Relative mRNA expression levels of ECs markers CDH5, CD31 and mesenchymal/ myofibroblastic cells markers vimentin, ACTA2, and FSP1 were assessed by qRT‐PCR. (d) The protein levels and densitometric quantification of VE‐cadherin: CD31: vimentin: α‐SMA, and FSP1 were determined by western blot analysis after the TGF‐β1 treatment 48 hr. Data were presented as mean ± SEM. *p < 0.05, **p < 0.01: and ***p < 0.001 versus corresponding Ctrl group. α‐SMA: α‐smooth muscle actin; DAPI: 4′:6‐diamidino‐2‐phenylindole; EndMT: endothelial–mesenchymal transition; FSP1: fibroblast‐specific protein‐1; HUVECs: human umbilical vein endothelial cells; mRNA: messenger RNA; qRT‐PCR: quantitative real‐time polymerase chain reaction; TGF‐β1: transforming growth factor beta1

We first evaluated the effect of TGF‐β1 on EndMT in cultured primary HUVECs. Exposure of HUVECs to TGF‐β1 for 48 hr caused an obvious alteration in cellular morphology from a polygonal, cobblestone‐like shape to a more spindle‐like, fibroblast shape (Figure 1a). Double fluorescence staining after the HUVEC treatment with TGF‐β1 showed increased expression of the fibroblast marker vimentin and reduced expression of the EC marker CD31, indicating that the cells underwent EndMT (Figure 1b). Quantitative real‐time PCR (qRT‐PCR) verified these observations. In the presence of TGF‐β1, the messenger RNA (mRNA) levels of VE‐cadherin and CD31 were greatly reduced, and vimentin, α‐SMA, and FSP1 levels were increased (Figure 1c). To further confirm that the EndMT was induced by TGF‐β1, the protein levels of the endothelial or mesenchymal markers were examined by western blot analysis. This analysis demonstrated that VE‐cadherin and CD31 were significantly downregulated and that the protein levels of vimentin, α‐SMA, and FSP1 were significantly upregulated after the TGF‐β1 treatment (Figure 1d). These results indicate that the TGF‐β1 treatment can induce EndMT in primary HUVECs.

### 3.2 The expression of Foxm1 is upregulated during TGF‐β1‐induced EndMT and regulates genes associated with EndMT

**Figure 2:**
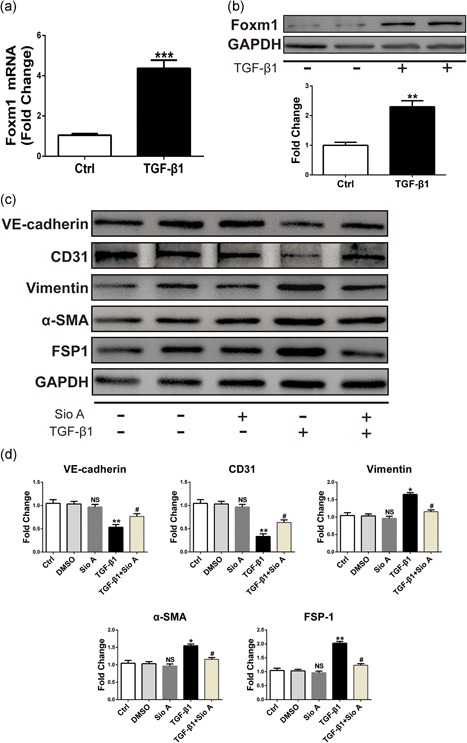
Increased Foxm1 expression was found after TGF‐β1 treatment and Foxm1 inhibitor Sio A can alleviate TGF‐β1‐induced EndMT in HUVECs. (a) Foxm1 mRNA expression assessed by qRT‐PCR in HUVECs exposed to control conditions (unstimulated) or TGF‐β1 for 48 hr. (b) Foxm1 protein level assessed by western blot under identical conditions. (c) The relative protein levels of VE‐cadherin: CD31: vimentin, α‐ SMA, and FSP1 were assessed by western blot. (d) d is the quantitative analysis of c. Data were presented as mean ± SEM. *p < 0.05, **p < 0.01: and ***p < 0.001 versus corresponding Ctrl group. ^#^ *p*<0.05 vs corresponding TGF‐β1 group. α‐SMA: α‐smooth muscle actin; EndMT: endothelial‐mesenchymal transition; Foxm1: forkhead box M1; FSP1: fibroblast‐specific protein‐1; HUVECs: human umbilical vein endothelial cells; mRNA: messenger RNA; qRT‐PCR: quantitative real‐time polymerase chain reaction; Sio A: Siomycin A; TGF‐β1: transforming growth factor beta1

**Figure 3:**
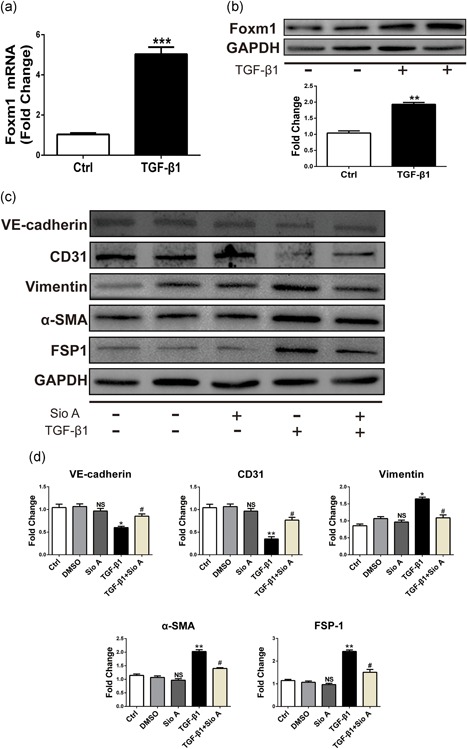
Increased Foxm1 expression was found after TGF‐β1 treatment and Foxm1 inhibitor Sio A can alleviate TGF‐β1‐induced EndMT in MAECs. (a) Foxm1 mRNA expression assessed by qRT‐PCR in MAECs exposed to control conditions (unstimulated) or TGF‐β1 for 48 hr. (b) Foxm1 protein level assessed by western blot under identical conditions. (c) The relative protein levels of VE‐cadherin: CD31: vimentin, α‐ SMA, and FSP1 were assessed by western blot. (d) d is the quantitative analysis of c. Data were presented as mean ± SEM. *p < 0.05, **p < 0.01: and ***p < 0.001 versus corresponding Ctrl group. ^#^ *p*<0.05 versus corresponding TGF‐β1 group. α‐SMA: α‐smooth muscle actin; EndMT: endothelial‐mesenchymal transition; Foxm1: forkhead box M1; FSP1: fibroblast‐specific protein‐1; MAECs: mouse aortic endothelial cells; mRNA: messenger RNA; qRT‐PCR: quantitative real‐time polymerase chain reaction; Sio A: Siomycin A; TGF‐β1: transforming growth factor beta1

Previous studies have shown that Foxm1 increased radiation‐induced pulmonary fibrosis by promoting EMT in alveolar epithelial cells. To identify a possible regulatory role of Foxm1 in the TGF‐β1‐induced EndMT in ECs, we analyzed the Foxm1 expression in TGF‐β1‐treated HUVECs and mouse aortic endothelial cells (MAECs). The qRT‐PCR showed increased expression of Foxm1 in TGF‐β1‐treated HUVECs (Figure 2a) and MAECs (Figure 3a). To confirm these results, western blot analysis was performed to test Foxm1 expression. As indicated in Figures 2b and 3b, the Foxm1 expression at the protein level was increased compared with the control group. To investigate the effect of Foxm1 on TGF‐β1‐induced EndMT, we tested the effect of Siomycin A (Sio A, an inhibitor of Foxm1 binding to DNA) at a concentration of 2.5 μM on the expression of Foxm1 target genes. To this end, we treated the HUVECs with Sio A and found that Sio A alone had no effect on EndMT. However, the Sio A was sufficient to suppress TGF‐β1‐induced EndMT. As shown in Figures 2c and 3c, the expression of fibroblast markers vimentin, α‐SMA, and FSP1 was significantly reduced compared with levels in the TGF‐β1 group. Meanwhile, inhibition of Foxm1 caused a significant increase in EC markers, VE‐cadherin, and CD31 in cells treated with TGF‐β1. Figures 2d and 3d show the quantitative analysis of EndMT‐related protein levels. Altogether, the loss of Foxm1 function and decreased expression of mesenchymal markers and increased level of endothelial VE‐cadherin and CD31 in TGF‐β1‐treated HUVECs likely contribute to decreased EndMT in Foxm1‐inhibited cells after the TGF‐β1 treatment.

### 3.3 Knockdown of Foxm1 with shRNA inhibits TGF‐β1‐induced EndMT and HUVEC cell migration

**Figure 4:**
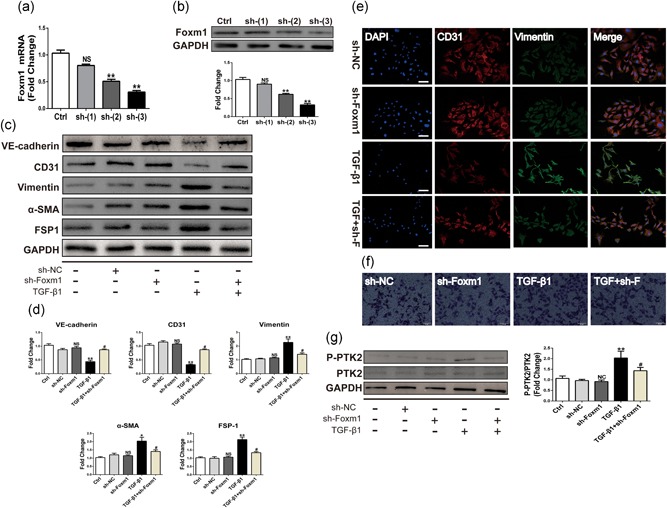
Silencing of Foxm1 with shRNA attenuated TGF‐β1‐induced EndMT and migration in HUVECs. (a,b) Knockdown efficiency of three different interference sequences of Foxm1 was verified by qRT‐PCR and western blot. (c) The relative protein levels of VE‐cadherin, CD31, vimentin, α‐SMA, and FSP1 were assessed by western blot. (d) d is the quantitative analysis of c. (e) Cells were analyzed by immunofluorescence for the expression of CD31 (red), vimentin (green), and nuclei (DAPI: blue). scale bar: 200 μm. (f) Silencing of Foxm1 ameliorated TGF‐β1‐induced cellular migration. scale bar, 50 μm. (g) Expression of phosphorylated PTK2 was evaluated by western blot and quantified relative to total PTK2 expression. Data were presented as mean ± SEM. *p < 0.05, **p < 0.01 versus corresponding Ctrl group. ^#^ *p*< 0.05 versus corresponding TGF‐β1 group. α‐SMA, α‐smooth muscle actin; DAPI, 4′,6‐diamidino‐2‐phenylindole; EndMT: endothelial‐mesenchymal transition; Foxm1: forkhead box M1; FSP1: fibroblast‐specific protein‐1; HUVECs: human umbilical vein endothelial cells; PTK2: protein tyrosine kinase 2; qRT‐PCR: quantitative real‐time polymerase chain reaction; shRNA: short hairpin RNA; TGF‐β1: transforming growth factor beta1

To further investigate the effect of Foxm1 on TGF‐β1‐induced EndMT, shRNA‐mediated transfection was used for 24 hr to downregulate Foxm1 followed by treatment with TGF‐β1 for 48 hr. We transfected HUVECs with three Foxm1‐specific shRNAs and confirmed gene knockdown efficiency by the qRT‐PCR 48 hr posttransfection. We found that sh‐Foxm1(3) has the most significant knockdown efficiency, which reaches 70% at the mRNA level (Figure 4a). This knockdown efficiency has also been verified at the protein level (Figure 4b). Therefore, the following experiments were performed using sh‐Foxm1(3). Compared with sh‐NC, Foxm1 shRNA significantly attenuated the TGF‐β1‐induced EndMT in HUVECs. As shown in Figure 4c,d, Foxm1 silencing reduced the expression of TGF‐β1‐induced mesenchymal markers vimentin, α‐SMA, and FSP1. In contrast, inhibition of Foxm1 caused a significant increase in EC markers VE‐cadherin and CD31 in HUVECs treated with TGF‐β1. In addition, increased endothelial marker CD31 and decreased mesenchymal marker vimentin in HUVECs transfected with Foxm1 shRNA were visualized using dual‐immunofluorescence staining with TGF‐β1 stimulation. Furthermore, as shown by Transwell experiments, sh‐Foxm1 suppressed the TGF‐β1‐induced migration of HUVECs. The above experiments demonstrated that targeting of Foxm1 can inhibit TGF‐β1‐induced EndMT process and cell migration.

### 3.4 Knockdown of Foxm1 with shRNA inhibits TGF‐β2‐induced EndMT in HMECs

**Figure 5:**
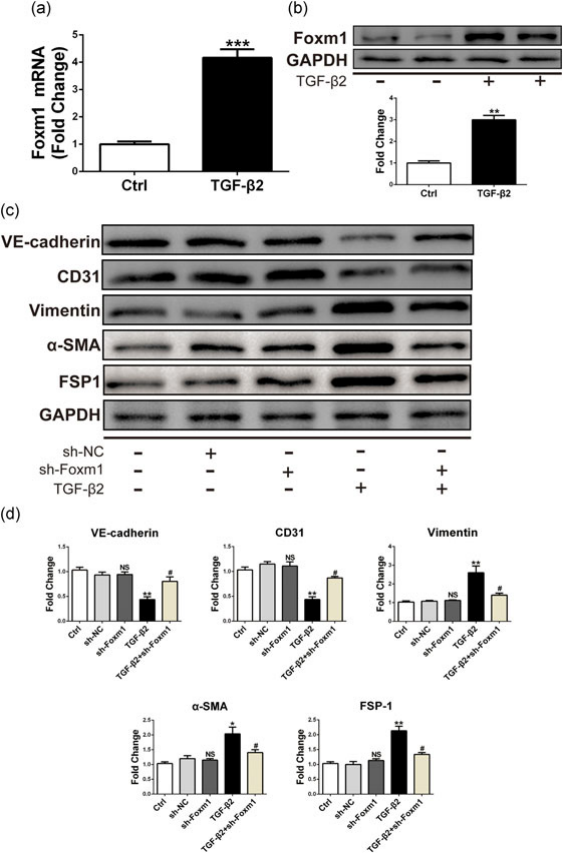
Increased Foxm1 expression was found after TGF‐β2 treatment and silencing of Foxm1 with shRNA can alleviate TGF‐β2‐induced EndMT in HMECs. (a) Foxm1 mRNA expression assessed by qRT‐PCR in HMECs exposed to control conditions (unstimulated) or TGF‐β2 for 48 hr. (b) Foxm1 protein level assessed by western blot under identical conditions. (c) The relative protein levels of VE‐cadherin, CD31: vimentin: α‐ SMA: and FSP1 were assessed by western blot. (d) d is the quantitative analysis of c. Data were presented as mean ± SEM. *p < 0.05, **p < 0.01, and ***p < 0.001 versus corresponding Ctrl group. ^#^ *p*<0.05 versus corresponding TGF‐β2 group. α‐SMA, α‐smooth muscle actin; EndMT: endothelial‐mesenchymal transition; Foxm1: forkhead box M1; FSP1: fibroblast‐specific protein‐1; HMECs: Human microvascular endothelial cells; mRNA: messenger RNA; qRT‐PCR: quantitative real‐time polymerase chain reaction; shRNA: short hairpin RNA; TGF‐β2, transforming growth factor beta2

Sabbineni et al. demonstrated that TGF‐β2 causes strong EndMT induction in HMECs (human microvascular endothelial cells) (Sabbineni et al., 2018). Therefore, we wanted to demonstrate the role of Foxm1 in TGF‐β2‐induced EndMT in HMEC cells. First, the qRT‐PCR showed increased expression of Foxm1 in TGF‐β2‐treated HMECs (Figure 5a). To further confirm this result, western blot analysis was performed to test Foxm1 expression. As shown in Figure 5b, the Foxm1 expression at the protein level was increased compared with that in the control group. To investigate the Foxm1 effect on TGF‐β2‐induced EndMT, shRNA‐mediated transfection was used for 24 hr to downregulate Foxm1 followed by treatment with TGF‐β2 for 48 hr. Compared with sh‐NC, Foxm1 shRNA significantly attenuated the TGF‐β2‐induced EndMT in HMECs. As shown in Figure 5c,d, Foxm1 silencing reduced the TGF‐β2‐induced expression of the mesenchymal markers vimentin, α‐SMA, and FSP1. Conversely, Foxm1 inhibition caused a significant increase in the EC markers VE‐cadherin and CD31 in HMECs treated with TGF‐β2. The above experiments demonstrated that targeting Foxm1 inhibits both TGF‐β1‐ and TGF‐β2‐induced EndMT in different ECs.

### 3.5 Overexpression of Foxm1 promotes EndMT in ECs

**Figure 6:**
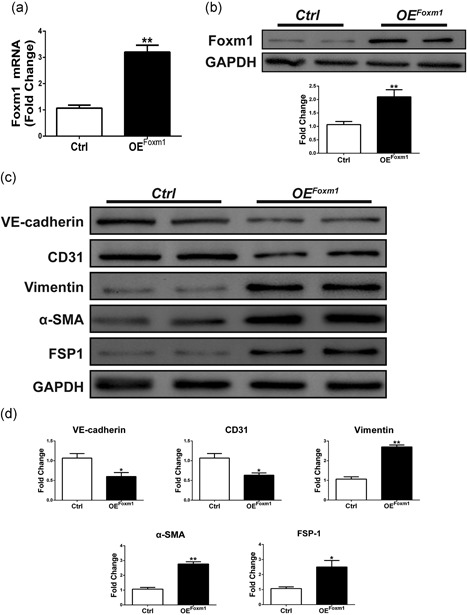
Foxm1 overexpression can induce EndMT in HUVECs. (a,b) qRT‐PCR and western blot analysis of expression of Foxm1 in cells transfected with FOXM1 overexpression plasmid for 48 hr. (c) The relative protein levels of VE‐cadherin, CD31: vimentin, α‐SMA: and FSP1 were assessed by western blot in cells transfected with FOXM1 overexpression plasmid for 48 hr. (d) d is the quantitative analysis of c. Data were presented as mean ± SEM. *p < 0.05: **p < 0.01 versus corresponding Ctrl group. α‐SMA: α‐smooth muscle actin; EndMT: endothelial‐mesenchymal transition; Foxm1: forkhead box M1; FSP1: fibroblast‐specific protein‐1; HUVECs: human umbilical vein endothelial cells; OE: overexpression; qRT‐PCR: quantitative real‐time polymerase chain reaction

To determine whether FOXM1 is sufficient to drive EndMT in HUVECs, we used lentivirus‐based Foxm1 overexpression. Overexpression of Foxm1 increased the Foxm1 mRNA level approximately three‐fold (Figure 6a) and increased protein expression approximately two‐fold (Figure 6b). Indeed, forced overexpression of Foxm1 resulted in changes in EndMT‐related protein levels. As shown in Figure 5c,d, the expression of VE‐cadherin and CD31 was significantly reduced, whereas the expression of mesenchymal‐related proteins, such as vimentin, α‐SMA, and FSP1, was significantly increased. Altogether, the Foxm1 overexpression and inhibition effects provided double verification that Foxm1 is indeed involved in the EndMT process.

### 3.6 Foxm1 regulates TGF‐β‐induced EndMT through the Smad2/3 signaling pathway and directly activates the Snail promoter

**Figure 7:**
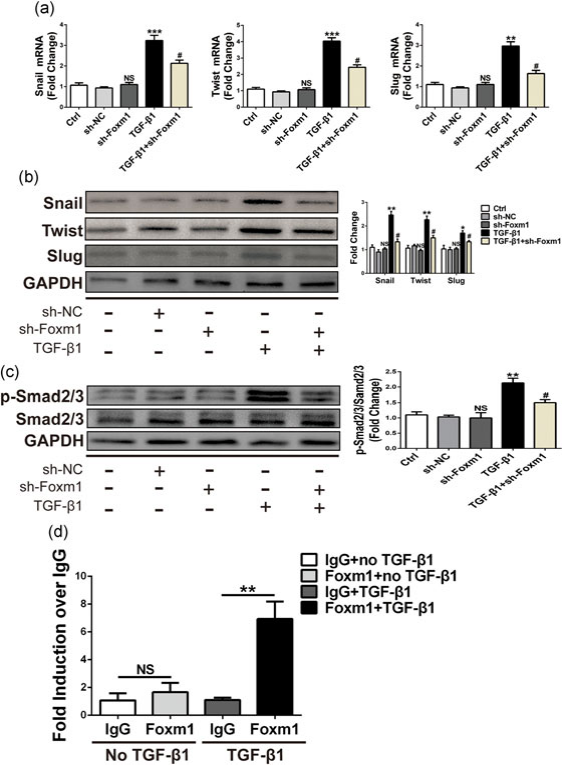
Foxm1 regulates the expression of EndMT‐related transcription factors and can directly activate the Snail1 promoter. (a,b) Representative EndMT‐related transcription factor mRNA (a) and protein (b) levels in HUVECs. (c) Representative western blot and quantitative results of the protein levels of p‐Smad2/3 and total Smad2/3 in the HUVECs of the indicated groups. (d) Foxm1 directly binds to the Snail1 promoter region after TGF‐β1 treatment. Data were presented as mean ± SEM. *p < 0.05, **p < 0.01, and ***p < 0.001 versus corresponding Ctrl group. ^#^ *p*<0.05 versus corresponding TGF‐β1 group. EndMT: endothelial–mesenchymal transition; Foxm1: forkhead box M1; HUVECs, human umbilical vein endothelial cells; mRNA, messenger RNA; qRT‐PCR: quantitative real‐time polymerase chain reaction; TGF‐β1: transforming growth factor beta1

**Figure 8:**
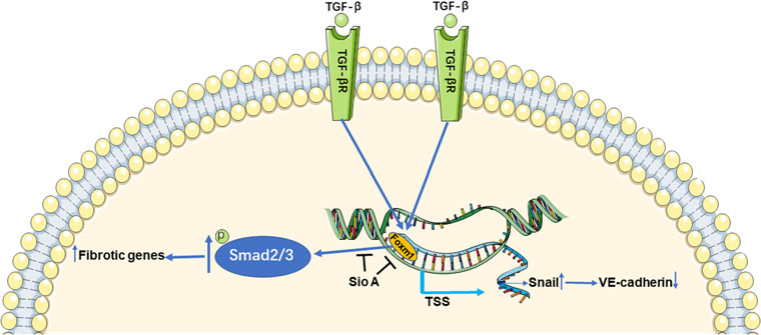
Diagrammatic sketch of the working hypothesis. Foxm1 promotes the TGF‐β1‐induced EndMT process in ECs through Smad2/3 signaling pathway and direct binding to and inducing the transcriptional activity of the *Snail* gene: an EndMT‐promoting transcription factor. ECs: endothelial cells; EndMT: endothelial‐mesenchymal transition; Foxm1: forkhead box M1; Sio A: Siomycin A; TGF‐β, transforming growth factor beta; TGF‐βR: transforming growth factor beta receptor; TSS: transcriptional start site

By the above‐described results, we have demonstrated that Foxm1 can regulate the EndMT process in ECs. Several EndMT‐promoting transcription factors, such as Snail, Slug, and Twist, have been implicated in EndMT (Sabbineni et al., 2018). Next, we wanted to examine whether Foxm1 regulated the EndMT process through these three transcription factors. We found that silencing Foxm1 can reverse increases in Snail, Slug, and Twist mRNA expression induced by TGF‐β1 stimulation (Figure 7a). This effect was also verified by western blot analysis (Figure 7b). Canonical Smad signaling appears to be involved in TGF‐β1‐induced EndMT. We also found that TGF‐β1 increased the expression levels of p‐Smad2/3 in HUVECs, whereas advanced knockdown of Foxm1 reduced the increase in TGF‐β1‐induced expression of this protein (Figure 7c). Furthermore, Balli et al. reported that the EndMT‐promoting transcription factor Snail was a direct transcriptional target of Foxm1 in alveolar epithelial cells (AECs). Foxm1 bound to and increased promoter activity of the *Snail* gene (Balli et al., 2013). Since Foxm1 induced Snail mRNA and protein in HUVECs, we wanted to investigate whether Snail was a direct transcriptional target of Foxm1. A potential Foxm1‐binding site was identified within the −1.0‐Kb promoter region of the human *Snail* gene. A chromatin immunoprecipitation (ChIP) assay was used to determine whether Foxm1 binds to the promoter region of the *Snail* gene in HUVECs. After the TGF‐β1 treatment, the specific binding of the Foxm1 protein to the Snail promoter DNA was increased (Figure 7d). Thus, Foxm1 directly bound to and induced the transcriptional activity of the *Snail* gene, indicating that *Snail* is a direct Foxm1 target, and Foxm1 influenced EndMT through *Snail*. In addition, Foxm1 might also partly regulate the expression of fibrosis‐related genes through the Smad signaling pathway (Figure 8).

## 4 DISCUSSION

Here, we have shown for the first time that Foxm1 promotes the TGF‐β‐induced EndMT process in ECs through the Smad2/3 signaling pathway and direct binding to and inducing the transcriptional activity of the *Snail* gene encoding an EndMT‐promoting transcription factor. Our results confirmed that TGF‐β‐exposed ECs could undergo EndMT and that Foxm1 was significantly upregulated in multiple ECs after the TGF‐β treatment. Using both molecular and pharmacological approaches to interrogate the functional roles of Foxm1, we found that Foxm1 inhibition prevented TGF‐β‐induced EndMT as well as cell migration. In contrast, lentivirus‐mediated overexpression of Foxm1 allowed EndMT to proceed despite the absence of TGF‐β in ECs. We have suggested that Foxm1 is indeed involved in the EndMT process by using both knockdown and overexpression approaches. We also found that Foxm1 regulates the primary mechanism of EndMT via the Smad2/3 pathway induced by TGF‐β and direct binding to the promoter region of the *Snail* gene. These results suggest that Foxm1 may be an important mediator of EndMT‐associated CF.

EndMT plays an important role in the pathogenesis of CF by associating with the emergence of fibroblasts of an EC origin (Zeisberg et al., 2007). During EndMT, several molecular and structural rearrangements take place leading to the cellular changes necessary to switch to a mesenchymal phenotype. EndMT results in ECs without cell–cell adhesion, with high migratory potential and with expression of specific mesenchymal cell markers, such as vimentin, α‐SMA, and FSP1 (Cooley et al., 2014). Concurrently, ECs undergoing EndMT lose the expression of characteristic surface EC markers, such as VE‐cadherin and CD31 (Figure 1). Zeisberg et al. demonstrated that TGFβ was implicated in a signaling pathway that stimulated EndMT in cardiac injury. TGF‐β induced ECs to undergo EndMT, which provided evidence of the EndMT role in aortic banded‐induced cardiac fibrosis (Zeisberg et al., 2007). In addition, Sabbineni et al. demonstrated that TGF‐β2 was the most potent inducer of EndMT (Sabbineni et al., 2018). Our results also demonstrate that Foxm1 plays an important role in TGF‐β2‐induced EndMT (Figure 6). Furthermore, EndMT significantly contributes to myocardial fibrosis in the human adult heart and animal disease models (Xu et al., 2015a). Development of technological innovations to better visualize the EndMT is a key for therapeutic targeting of EndMT in human CF.

EndMT is related to the more widely known mechanism of EMT. There is strong evidence that Foxm1 is a key regulator of EMT, and it is well recognized as a driver of transcriptional activation of EMT‐regulator expression as well as the expression of typical mesenchymal markers (Huang et al., 2012). To our knowledge, our data are the first to establish the importance of Foxm1 in EndMT‐mediated fibrosis. The antifibrotic function of Foxm1 has been reported in cardiac hypertrophy and fibrosis, diabetes, and pulmonary fibrosis (Penke et al., 2018; Yang et al., 2016). This study showed that Foxm1 expression was upregulated in TGF‐β1‐induced EndMT and that Foxm1 knockdown could inhibit TGF‐β1‐induced EndMT and cell migration. Balli et al. reported that one of the Foxm1 roles during lung fibrosis was to induce EMT through direct transcriptional activation of Snail and promote pulmonary inflammation through increased expression of inflammatory mediators (Balli et al., 2013). In addition, a previous study showed that Foxm1 overexpression significantly polymerizes actin assembly and impairs E‐cadherin expression, resulting in EMT, and metastasis in a xenograft mouse model, whereas Foxm1 knockdown has the opposite effect (Zhang et al., 2017). The above results demonstrated that Foxm1 is indeed involved in the EMT process. However, the potential mechanism of TGF‐β1 regulation of the EndMT through Foxm1 expression is still not understood.

Both in vitro and in vivo studies have shown that TGF‐β plays a central role in endocardial EndMT. Sabbineni et al. proved that all three TGF‐β isoforms (TGF‐β1, TGF‐β2, and TGF‐β3) induced EndMT in HMECs after 72 hr, which resulted in phosphorylation of Smad2 and Smad3 (Sabbineni et al., 2018). Activated Smad2 and Smad3 form a complex with Smad4. It has also been reported that FOXM1 interacts with Smad3 to sustain activation of the Smad2/Smad3/Smad4 complex in the nucleus (Xue et al., 2014). The complex translocates to the nucleus and activates ECM associated genes, as well as *Snail* 1, 2, and *Twist* genes, which triggers a cascade of signaling pathways that culminate in EndMT (Sabbineni et al., 2018; Yoshimatsu & Watabe, 2011). Cooley et al. (2014) have shown that the TGF‐β‐Smad2/3‐Slug signaling pathway plays a pivotal role in regulating vein graft EndMT, with in vivo reduction of TGF‐β signaling decreasing both neointimal formation and the relative contribution of endothelial lineage‐derived cells to the neointima. Interestingly, the two most recent studies indicate the unique role of the Smad1/5 pathway in EndMT (Ramachandran et al., 2018; Sniegon, Priess, Heger, Schulz, & Euler, 2017), in addition to the observed role of the Smad2/3–Snail pathway. All studies listed above suggested that TGF‐β signaling plays a critical role in EndMT. Our data showed that TGF‐β1 increased phosphorylation of Smad2/3 in HUVECs, whereas inhibition of Foxm1 attenuated this activation.

Loss of VE‐cadherin is a hallmark of EndMT. In our study, Foxm1 knockdown with shRNA resulted in the loss of EndMT‐related transcription factors after the TGF‐β1 treatment. Previous studies demonstrated that the loss of VE‐cadherin was mediated by the inhibitor binding to the E‐box sequences within the VE‐cadherin promoter (Hennig, Lowrick, Birchmeier, & Behrens, 1996). Snail represses VE‐cadherin expression through direct binding to these E‐boxes (Cano et al., 2000). Balli et al. reported that Foxm1 bound to and increased promoter activity of the *Snail* gene in alveolar epithelial cells (Balli et al., 2013). Consistent with the important role of Snail in EndMT, the present studies demonstrated that Foxm1 induced Snail mRNA and protein in HUVECs. We investigated whether Snail was a direct transcriptional target of Foxm1 in HUVECs. In the context of endogenous Snail promoter, TGF‐β1 increased Foxm1 binding to the Snail promoter DNA as demonstrated by ChIP assay, suggesting that there was crosstalk between TGF‐β1 signaling and Foxm1 in the regulation of the Snail promoter. The finding that Foxm1 directly bound to and increased the activity of the Snail promoter demonstrates that Snail is a direct transcriptional target of Foxm1 providing a mechanism by which Foxm1 induces EndMT and potentially contributes to TGF‐β1‐induced CF.

In conclusion, Foxm1 promotes TGF‐β1‐induced EndMT via direct activation of the Smad2/3 signaling pathway and binding to the Snail promoter DNA. Even though TGF‐β has been identified as the single most important growth factor that can induce EndMT, there may be other signaling pathways, such as Wnt and MAPK, that can also regulate EndMT (Gonzalez & Medici, 2014). Whether Foxm1 regulates EndMT through other signaling pathways needs further investigation. Our findings suggest that Foxm1 might be a prospective target for TGF‐β1‐induced EndMT and a potential target in CF therapy.

## ETHICAL STATEMENT

The local ethics committee approved the human research. The human research was performed in compliance with the Declaration of Helsinki. Animal studies have been approved by the ethics committee of Xinhua Hospital affiliated to Shanghai Jiao Tong University School of Medicine and have therefore been performed in accordance with the ethical standards laid down in the 1964 Declaration of Helsinki and its later amendments.

## CONFLICTS OF INTEREST

The authors declare that they have no conflicts of interest.

## Supporting information

Supporting information
