# Supplementary material for: Foxm1 is a critical driver of TGF‐β‐induced EndMT in endothelial cells through Smad2/3 and binds to the Snail promoter
Source: J Cell Physiol. 2018 Oct 30;234(6):9052–64. doi: 10.1002/jcp.27583 (PMC6686160; doi:10.1002/jcp.27583)
Supplement: Supplementary file 1 — Supporting information [file JCP-234-9052-s001.docx]

**Table S1** Tables of sequences and downstream primers for genes analyzed by qRT-PCR(h-human, m-mouse).

| Gene | Forward Primer | Reverse Primer |
| --- | --- | --- |
| Foxm1(h) | 5’-gatctgcgagattttggtacac-3’ | 5’- ctgcagaagaaagaggagctat-3’ |
| CDH5(h) | 5’-aaagaatccattgtgcaagtcc-3’ | 5’-cgtgttatcgtgattatccgtg-3’ |
| CD31(h) | 5’-gacatgaagagcctgccgga-3’ | 5’-ggcttgacgtgagaggtggt-3’ |
| Vimentin(h) | 5’-tacgaggaggagatgcggga-3’ | 5’-catgatgtcctcggccaggt-3’ |
| ACTA2(h) | 5’-ctcgtgtgcgacaatggctc-3’ | 5’-cgtcgcccacgtaggaatct-3’ |
| FSP1(h) | 5’-tgatgagcaacttggacagcaacag-3’ | 5’-catggcgatgcaggacaggaag-3’ |
| Snail(h) | 5’-aatccagagtttaccttccagc-3’ | 5’-gaagtagaggagaaggacgaag-3’ |
| Twist(h) | 5’-gtacatcgacttcctctaccag-3’ | 5’-catcctccagaccgagaag-3’ |
| Slug(h) | 5’-ctgtgacaaggaatatgtgagc-3’ | 5’-ctaatgtgtccttgaagcaacc-3’ |
| GAPDH(h) | 5’-ccagaacatcatccctgcct-3’ | 5’-cctgcttcaccaccttcttg-3’ |
| Foxm1(m) | 5’-acattggaccaagtgtttaagc-3 | 5’-tttctatggagctcaggattgg-3 |
| CDH5(m) | 5’- cccactatccgatacgaatacc-3 | 5’-atccacatctaggacgttgatg -3 |
| CD31(m) | 5’-cacaacaaacaagctagcaaga -3 | 5’-tttggctgcaactattaaggtg-3 |
| Vimentin(m) | 5’-ttgccgttgaagctgctaactacc -3 | 5’-aatcctgctctcctcgccttcc -3 |
| ACTA2(m) | 5’-gtcccagacatcagggagtaa-3’ | 5’-tcggatacttcagcgtcagga-3’ |
| FSP1(m) | 5’-caaagagggtgacaagttcaag-3’ | 5’-cagtactcctggaagtcaactt-3’ |
| GAPDH(m) | 5’-aggtcggtgtgaacggatttg-3’ | 5’-tgtagaccatgtagttgaggtca-3’ |

Note: qRT-PCR, quantitative real‐time polymerase chain reaction.
